# Nocebo Effect in Randomized Clinical Trials of Antidepressants in Children and Adolescents: Systematic Review and Meta-Analysis

**DOI:** 10.3389/fnbeh.2014.00375

**Published:** 2014-11-03

**Authors:** Johanna Carolina Rojas-Mirquez, Milton Jose Max Rodriguez-Zuñiga, Francisco Javier Bonilla-Escobar, Herney Andres Garcia-Perdomo, Mike Petkov, Lino Becerra, David Borsook, Clas Linnman

**Affiliations:** ^1^The Pain and Analgesia Imaging Neuroscience (P.A.I.N.) Group, Department of Anesthesiology, Perioperative and Pain Medicine, The Center for Pain and the Brain, Boston Children’s Hospital, Harvard Medical School, Waltham, MA, USA; ^2^Grupo de Epidemiología del Trauma y las Lesiones, Universidad del Valle, Cali, Colombia; ^3^Instituto Cisalva, School of Public Health, Universidad del Valle, Cali, Colombia; ^4^Cochrane Center, Universidad del Valle, Cali, Colombia

**Keywords:** nocebo, children, adolescents, antidepressants, meta-analysis

## Abstract

**Objective**: To compare the incidence of adverse events between active and placebo arms of randomized clinical trials in depressive children and adolescents (C&A) with antidepressant treatments, in order to look for similarities in both groups that allow to establish a possible nocebo effect.

**Methods**: Systematic search strategy (January 1974–March 2013) in electronic databases, conference abstracts, and reference list of systematic reviews and included studies to identify parallel randomized placebo-controlled trials of antidepressants in C&A (<19 years) with major depressive disorder, and one or more interventions of any orally administered antidepressant. The pooled adverse events were calculated based on a fixed-effect model and statistical analysis involved the risk ratio (RR) of adverse events, with 95% confidence intervals (95% CI).

**Results**: Sixteen studies were included in the review, of which seven studies with a sample of 1911 patients had data to include in the meta-analysis. There was similar risk for the incidence of adverse events between non-active and active group (global RR 1.04, 95% CI: 0.97–1.11).

**Conclusion**: Depressive C&A allocated to placebo or active group had similar risk to develop adverse events. These similarities in both groups are attributed to the nocebo effect. It is of note that defining “nocebo” effects is challenging in clinical populations because adverse effects may be attributed to the intervention or may be manifestation of the disease itself. The inclusion of a no-treatment arm may be warranted. Nocebo effects are likely when adverse events of placebo mimic the adverse events of active treatment, as was the case here.

## Introduction

The use of antidepressants for children and adolescents (C&A) is still a matter of debate due to questionable efficacy and risk of adverse events. The efficacy of these medications appears to be influenced by patient such as function indication, age, chronicity, and study characteristics (Bridge et al., 2007). Negative effects of placebo – nocebo effects (NE) – occur in 25% of patients (Hauser et al., 2012a). NE can be induced by negative expectation, suggestion, and previous experiences, and are influenced by psychological characteristics as well as therapy situation and context (Wells and Kaptchuk, 2012; Ciaramella et al., 2013), which can influence the expectations of patient, the clinician, and other involved parties.

Systematic reviews have proved the efficacy of antidepressants compared to placebo in adults with major depressive disorder (MDD) (Undurraga and Baldessarini, 2012). The adverse effect profiles between different therapy groups such as selective serotonin receptor inhibitors (SSRIs) and tricycle antidepressants (TCAs) are prone to systematic expectation influences in both patients and investigators (Gracely et al., 1985; Mora et al., 2011). In C&A, the effectiveness of antidepressant drugs is a controversial topic (Hazell, 2009) since there is an important incidence of AE and suicide-related events using SSRIs. Hence, these drugs have received a “black-box” warning by the Food and Drug Administration and the Medicines and Healthcare products Regulatory Agency cautions physicians on their use in C&A (Hetrick et al., 2012).

Defining “nocebo” effects is challenging in clinical populations. An adverse effect (such as dry mouth, loose stool, frequent urinations, or insomnia) may be a “nocebo” or it may be somatic manifestations of the disease itself. Additionally, factors related with NE have been identified in the pediatric population that may lead to confusion, such as divergent behaviors of the disease and parental/cultural influence (Fernandes et al., 2008). The nocebo profile seems to parallel drug side effects in adults (Brambilla et al., 2005). In the context of a randomized clinical trial, in order to assess a true placebo or nocebo effect, the non-active drug should ideally be compared to a no-treatment group. True placebo response would be symptom improvements in the non-active treatment arm that go above and beyond spontaneous remission in the no-treatment group. Likewise, true nocebo responses are adverse effects that go above and beyond symptoms in the no-treatment group. An unfortunate methodological fact is that very few RCT’s include such a no-treatment group. Thus, we call adverse events in non-active arms “nocebo-like effects,” with the caveat that such effects may in part be spontaneous symptoms and manifestations of disease rather than true NE. This meta-analysis aims to compare the incidence of adverse events between active and placebo arms of randomized clinical trials in depressive C&A with SSRI, TCA, and SSNRI treatments, in order to look for similarities in adverse effect profiles in active and placebo arms.

## Materials and Methods

We conducted a systematic review and meta-analysis of antidepressant RCTs in C&A with MDD to describe and compare the AE in placebo and treatment arms. This study was conducted according to the recommendations of the Cochrane Collaboration (Higgins and Green, 2011a) and the PRISMA Statement (Moher et al., 2009), PROSPERO code CRD42013004638 (Rojas-Mirquez et al., 2013).

### Eligibility criteria

#### Studies

We included parallel, placebo-controlled RCTs of antidepressants in depressed C&A conducted between 1/1/1974 and 3/31/2013. Other types of RCTs and those that assessed other condition simultaneously were excluded. No language restrictions were imposed.

#### Participants

Female and male children (6–12 years) (National Center for Biotechnology Information, 2013a) and/or adolescents (13–18 years) (National Center for Biotechnology Information, 2013b) with MDD diagnosed with specific criteria. There were no preferences in any other demographic characteristic of participants.

#### Interventions

RCTs that compared placebo with one or more orally administered antidepressant drugs. There was no restriction of dosage, frequency, or duration of the treatment.

#### Outcomes

The primary outcome measure was the incidence of AE in placebo and treatment groups looking for similarities in both groups that allow to establish a possible NE. The secondary outcome was the efficacy of antidepressant treatment for C&A with MDD.

### Information sources and search strategy

We designed a search strategy for RCTs published in PubMed, CENTRAL, EMBASE, and BIREME. The search strategy was specific for each database and includes a combination of the medical subject headings and free text terms (see Table 1). *Clinicaltrials.gov* for RCTs and *biosis.org* for conference abstracts were used to find additional studies, as well as reference lists of selected articles, reviews, and previous meta-analyses.

**Table 1:** **Study and patients characteristics of RCTs included into the analysis**.

Author	Therapy group	FDA approval	Therapy duration (days)	Population	Sample size	Patients on placebo (*n*)	Patients on therapy group (*n*)	Dropouts (*n*)	Efficacy outcome measure tool	Baseline measure (mean)		Postreatment (mean)		Δ Efficacy		Assessment strategy for AEs	AEs placebo (*n*)	AEs therapy group (*n*)
										Placebo	Therapy	Placebo	Therapy	Placebo	Therapy	
Berard et al. (2006)	Paroxetine	No	84	Adole- scents	286	99	187	90	MADRS	25.9	25.9	13.1	12.3	12.8	13.6	Structured	56	119
Kye et al. (1996)	Amitriptyline	No	56	Adole- scents	31	13	18	9	HAM-D	13.2	12	8.8	8	4.4	4	Structured	NR	NR
Geller et al. (1992)	Nortriptyline	No	56	Children	60	29	31	10	CDRS-R	49.6	49.9	32	32.9	17.6	17	Structured	NR	NR
Keller et al. (2001)	(1): Paroxetine	No	56	Adole- scents	275	87	(1): 93	86	HAM-D	18.97	(1): 18.98	9.88	(1): 8.24	9.09	(1): 10.74	Observations	NR	NR
	(2): Imipramine						(2): 95				(2): 18.11		(2): 9.2		(2): 8.91	
Wagner et al. (2004)	Citalopram	No	56	Both	178	85	93	36	CDRS-R	57.8	58.8	41.8	37.8			Combination	NR	NR
GlaxoSmithKline (2011)	Paroxetine	No	56	Both	56	27	29	7	CDRS-R	NR	NR	−11.9	−16.5	11.9	16.5	Structured	9	9
Emslie et al. (2009)	Escitalopram	No	56	Adole- scents	316	158	158	53	CDRS-R	56	57.6	37.2	35.5	18.8	22.1	Combination	118	121
Simeon et al. (1990)	Paroxetine	No	49	Adole- scents	40	20	20	10	HAM-D	NR	NR	NR	NR			Not stated	NR	NR
Almeida-Montes (2005)	Fluoxetine	Yes	30	Both	23	11	12	7	DSR-S	NR	NR	NR	NR			Structured	NR	NR
Wagner et al. (2006)	Escitalopram	No	56	Both	268	136	132	51	CDRS-R	56.6	54.5	36.4	32.6	20.2		Combination	90	90
Eli Lilly and Company (2013a)	(1): Duloxetine	No	70	Both	337	103	(1): 117	72	CDRS-R	60.2	(1): 59.2	35.9	(1): 34.9	24.3	(1): 24.3	Structured	68	(1): 70
	(2): Fluoxetine						(2): 117				(2): 58.8		(2): 35.1		(2): 23.7			(2): 72
Kutcher et al. (1994)	Desipramine	No	42	Adole- scents	60	30	30	18	CDRS-R	23.77	22.63	13.42	12.68			Structured	NR	NR
Wagner et al. (2003)	Sertraline	No	70	Both	376	187	189	77	CDRS-R	64.6	64.3	38.77	34.06			Combination	NR	NR
Emslie et al. (2002)	Fluoxetine	yes	56	Both	219	110	109	61	CDRS-R	55.1	57.1	40.2	35.1	14.9	22	Combination	NR	NR
Eli Lilly and Company (2013b)	(1): Duloxetine 60 mg	No	70	Both	463	122	(1): 108	138	CDRS-R	58.2	(1): 59.3	36.6	(1): 35.4	21.6	(1): 23.9	Structured	71	(1): 76
	(2): Duloxetine 30 mg						(2): 116				(2): 11		(2): NR		(2): NR			(2): 66
	(3): Fluoxetine 30 mg						(3): 117				(3): 57.9		(3): NR		(3): NR			(3): 69
Emslie et al. (2006)	Paroxetine	No	56	Both	206	102	104	54	CDRS-R	62.6	60.7	39.2	38.1	23.4	22.6	Spontaneous reports	62	71

*(1), Arm 1; (2), Arm 2; (3), Arm 3*.

*FDA, Food and Drug Administration; *n*, number of patients; Δ, difference between baseline and post-treatment values on depression scales; AEs, adverse events; MADRS, Montgomery–Asberg Depression Rating Scale; HAM-D, Hamilton Depression Rating Scale; CDRS-R, Children’s Depression Rating Scale-Revised; DSR-S, Depression Self-Rating Scale; NR, no report; RCTs, randomized clinical trials*.

### Study selection

Two investigators (Johanna Carolina Rojas-Mirquez, Milton Jose Max Rodriguez-Zuñiga) independently screened the titles and abstracts to determine the potential usefulness of the articles. Eligibility criteria were applied to the full text articles during the final selection. We resolved disagreements by consensus and by a third reviewer (Herney Andres Garcia-Perdomo).

### Data collection process

All data were collected independently by two authors using a standardized data extraction sheet in Epi-Info™ 7.0 software (Centers for Disease Control and Prevention, CDC, Atlanta, GA, USA). An independent reviewer (Francisco Javier Bonilla-Escobar) confirmed all data entries and checked at least twice for completeness and accuracy.

### Data items

We extracted variables related with characteristics of the article, study design, patient’s data, and AE. All types of adverse events were included since authors of included clinical trials did not provide detail on the nature of adverse event reported. When data were not available, this was noted.

### Risk of bias in individual studies and across them

The Cochrane Collaboration risk of bias tool (Higgins et al., 2011; Sterne and Moher, 2011) was used independently by two researchers (Johanna Carolina Rojas-Mirquez, Milton Jose Max Rodriguez-Zuñiga). Disagreements were solved by consensus. A “Risk of bias table” and a risk of bias summary were edited using Review Manager Software Version 5.1^®^ (RevMan) to illustrate the judgments for each study.

### Quality assessment of AE

A Cochrane tool to assess quality and report of AE was used to evaluate the methodology that researchers used as well as the quality of the reports for published studies (Higgins and Green, 2011b).

### Synthesis of results

The risk ratio (RR) was the effect measured of the primary outcome, with 95% confidence intervals (95% CI). The efficacy of antidepressant treatment was defined as the standardized mean difference (SMD) between baseline and post-treatment values on depression scales. AE were only evaluated over the course of the intervention.

Included trials were characterized with descriptive statistics and median with 25th–75th percentiles (p25–p75) such as central tendency and dispersion measures. Quantitative data were analyzed with non-parametric tests, due to their non-normal distribution assessed by Shapiro–Wilk method. The confidence level was 95%, and these analyses were performed on Stata13^®^ (College Station, TX, USA: StataCorp LP).

Heterogeneity between trials was assessed through the *I*^2^ statistic, which indicates the percentage of variation in the effect size estimate attributable to heterogeneity rather than sampling error (*I*^2^ value greater or equal to 50% represents heterogeneity) (Higgins et al., 2003). The pooled AE and efficacy on both groups were calculated based on a fixed and random effect model, respectively, taking into account the heterogeneity of the studies. If studies show heterogeneity, results are based on a random effect model, and if studies are homogeneous, their results are based on a fixed-effect model (Bollen and Brand, 2010). Results are reported as forest plots showing the effect size of all the included studies with 95% CI.

#### Subgroup analysis

They were performed on the basis of age category (C&A) and drug category (SSRIs, NRIs, and TCAs) through RevMan^®^.

#### Sensitivity analysis

The relative RR was used for all primary analyses, and the Mantel–Haenszel random effects model was used for sensitivity analysis (Der-Simonian and Laird, 1986). We undertook the sensitivity analysis based on the exclusion of each one of the trials, as well as the unpublished and the smallest trials.

### Missing data

All data were used in the systematic review. Corresponding authors were contacted in the cases of missing data. The pooled available data were used for the meta-analysis.

## Results

### Study selection

**Figure 1:**
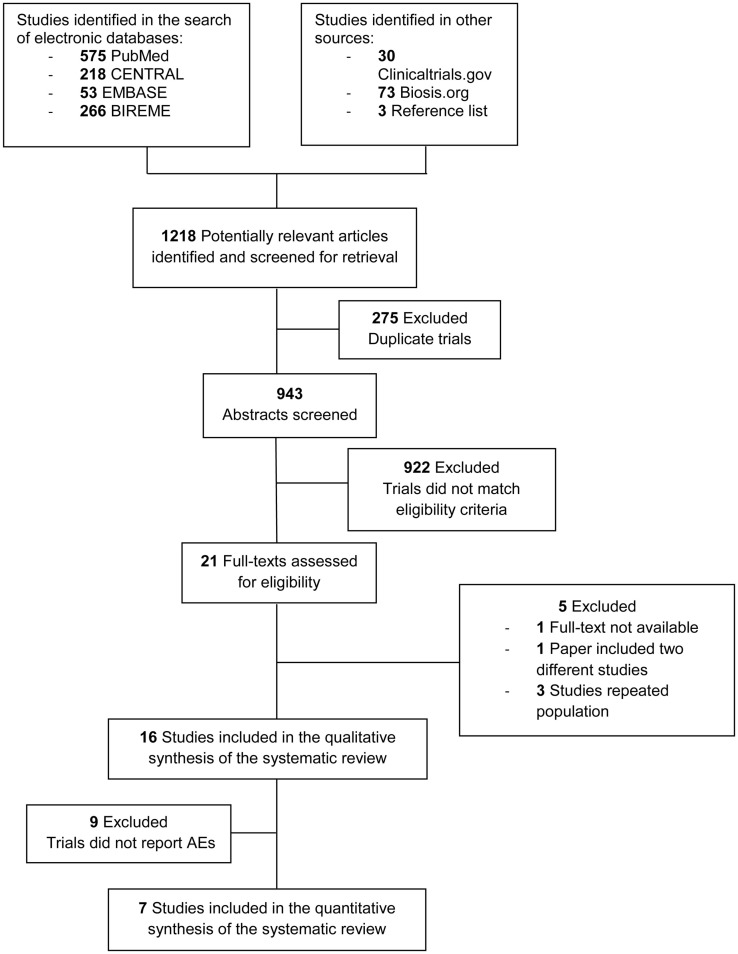
**Flow diagram on numbers of publications screened and included**. AEs, adverse events.

We identified 1018 studies. The final sample size meeting all inclusion criteria consisted of 16 studies performed in C&A with MDD, whereof seven studies met the criteria for quantitative analysis. Selection process and characteristics of excluded studies are detailed in Figure 1.

### Study characteristics

We analyzed 16 studies with a total of 3194 patients. Of those, 1319 were randomized to non-active and 1875 to an active group. The study sample sizes ranged from 23 to 463 patients. Thirteen were published in journals, while the rest were studies available on a clinical trial register for unpublished data (see Table 1).

In the age group analysis, nine out of 16 studies were carried out in both age groups (C&A), six trials were performed just in adolescents and one trial included only children (Geller et al., 1992). 313 children and 678 adolescents were included to non-active and 319 and 869 to an active drug, with no difference in proportions of children allocated to non-active or to an active arm by pharmacologic group (*p* = 0.17); however, there were significantly more adolescents in the SSNRI trials than in TCA trials (*p* = 0.002). The median age for all included participants was 13 (10–16), and it was similar for participants in the non-active and active group (*p* = 0.67).

All studies included both genders. In the 14 trials that reported the participants’ gender (3389 patients), 54% were female. Of those, 40% were allocated in non-active and 60% in an active group (*p* = 0.68). There was a significant difference between the proportion of males and females by pharmacologic group, with a higher proportion of males in the TCA studies and a majority of females in SSRI and SNRI studies (*p* = 0.0074).

Thirteen out of 16 trials assessed a SSRI in at least one of its arms, two a SNRI and four a TCA. Not including non-active arms, 13 studies had one active intervention arm, two trials had two active intervention arms (Keller et al., 2001; Eli Lilly and Company, 2013a), and one trial had three arms (Eli Lilly and Company, 2013b). There were 10 different drugs used in the active arms, with five paroxetine trials (Simeon et al., 1990; Keller et al., 2001; Berard et al., 2006; Emslie et al., 2006; GlaxoSmithKline, 2011), four fluoxetine (Emslie et al., 2002; Almeida-Montes, 2005; Eli Lilly and Company, 2013a,b), three duloxetine (Eli Lilly and Company, 2013a,b), two escitalopram trials (Wagner et al., 2006; Emslie et al., 2009), and single studies of imipramine (Keller et al., 2001), amitriptyline (Kye et al., 1996), nortriptyline (Geller et al., 1992), citalopram (Wagner et al., 2004), desipramine (Kutcher et al., 1994), and sertraline (Wagner et al., 2003).

A pharmaceutical company sponsored 13 out of the 16 trials, one was sponsored by the National Institute of Mental Health (Geller et al., 1992), one by the Nordic Merrell Dow Research and the Ontario Mental Health Foundation (Kutcher et al., 1994), and one did not provide information about the sponsor (Simeon et al., 1990).

The median intervention period had 56 (56–63) days of exposure to a drug, with 56 (30–84) days to SSRI, 70 (70–70) to SNRI, and 56 (42–56) days to TCAs, without significant differences (*p* = 0.11).

All trials used more than one tool for depression diagnosis with 12 out of 16 trials used the DSM-IV (Diagnostic and Statistical Manual of Mental Disorders, Fourth Edition), 10 using the Children Depression Scales-Revised (CDS-R), and 8 using the Kiddie-Schedule for affective disorders and schizophrenia for school-age children (K-SADS-P). All the trials assessed the efficacy of the drug as the primary outcome. The most frequent primary outcome measure tool used was CDRS-R in 11 trials, while three used Hamilton Depression Rating Scale, one used the Montgomery and Asberg Depression Rating Scale (MADRS) (Berard et al., 2006), and the Depression Self-Rating Scale (DSR-S), respectively (Almeida-Montes, 2005).

The total number of dropouts was 779, whereof 63% were allocated in the active group. The total rate of discontinuation was 22% with median discontinuation rate for placebo and active groups of 20% (11–38) and 25% (14–43), respectively. There were no statistically significant difference (*p* = 0.06), nor by pharmacologic group (*p* = 0.93).

### Risk of bias within and across studies

**Figure 2:**
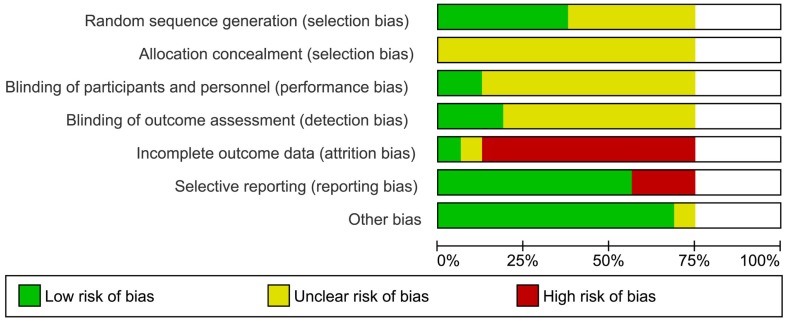
**Risk of bias across all included studies**.

The risk of bias summary was performed to show how each study was assessed for each domain (see Figure S1 in Supplementary Material). Around 90% of the trials had high risk of attrition bias, related to withdrawals and dropouts. All trials had an unclear risk of selection bias, associated to the lack of information about the allocation concealment process. Nearly 85% of trials had low risk of other bias, considering the comparable condition of the arms in their baseline characteristics. In all, 75% of studies were unclear about the masking method, although all trials were defined as double blinded (Figure 2).

### Quality assessment on conduct and report of AE

Cochrane’s tool was applied to 13 published studies, which provided the necessary information in order to do a judgment regarding the conduct and report of adverse events. The most frequent AE evaluation strategy was structured assessment (eight out of 16), using weekly scales, checklist, and questionnaires. The combination of assessment methods (five out of 16) consisted in spontaneous reports, physical examination of patients in each visit, and observation by investigators, while two trials used either spontaneous report of symptoms by the patient or observation and physical examination by the care professional in charge. One trial did not provide information on its assessment strategy for AE (Simeon et al., 1990). Of the seven studies included in the quantitative analysis, four used a structured method, either weekly AE monitoring or systematic assessment of AE, two used a combination that included spontaneous report and observation by investigators and one included study used the spontaneous report by patients (see Table S1 in Supplementary Material) (Emslie et al., 2006).

### Results of individual studies and synthesis of results

The most frequents AE were headache (RR 1.22, 95% CI 0.87–1.71), nausea (RR 1.88, 95% CI 1.44–2.45), dizziness (RR 2.04, 95% CI 1.41–2.96), abdominal pain (RR 1.18, 95% CI 0.81–1.72), vomiting (RR 1.58, 95% CI 1.07–2.34), insomnia (RR 2.16, 95% CI 1.42–3.27), somnolence (RR 1.51, 95% CI 0.78–2.92), and decrease appetite (RR 1.58, 95% CI 0.95–2.61); these are reported according to the rank of frequency of AE in placebo groups. Of the above symptoms, nausea, vomiting, insomnia, and dizziness had more significant risk to be present in patients taking antidepressants, compared to non-active treatment (see Figure S2 in Supplementary Material).

#### Nocebo effects

**Figure 3:**
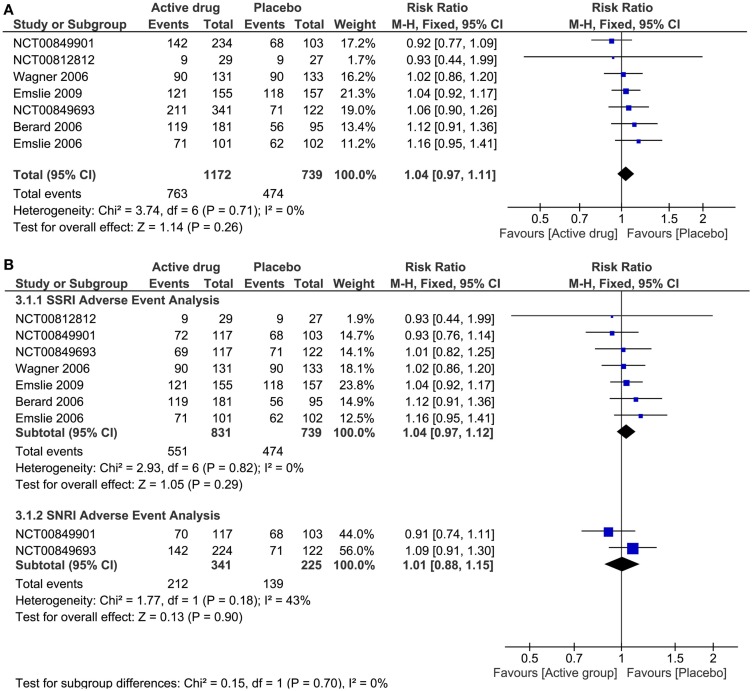
**Nocebo effect of antidepressant treatment**. **(A)** Forest plot of comparison: antidepressant treatment versus placebo; outcome: adverse events. **(B)** Forest plot of subgroup analysis: adverse event risk of placebo versus SSRI or SNRI. CI, confidence interval; SSRI, selective serotonin reuptake inhibitors; SNRI, serotonin-norepinephrine reuptake inhibitors.

The pooled effect estimate of NE was calculated with seven out of 16 trials that reported the number of patients who presented adverse events for each intervention group (1911 patients, 39% were in non-active group). The heterogeneity for the adverse effects’ RR was low (*I*^2^ = 0%), and there was no increased risk in developing them in patients allocated to an active drug and those allocated to non-active (RR 1.04, 95% CI 0.97–1.11). All the trials had similar risk to present AE in patients taking an active drug or taking placebo (Figure 3A). Table S2 in Supplementary Material shows the five most frequent AE for each one of the pharmacological group of antidepressants, and those that presented higher risk of development in non-active groups. Headache and nausea were the symptoms presented more frequently in patients allocated in non-active groups compared with any active treatment group, with proportions of 14 and 7%, respectively.

#### SSRIs trials

Following headache and nausea, nasopharyngitis, abdominal pain, and vomiting were the most common AE presented by patients of non-active groups (see Table S2 in Supplementary Material). Nasopharyngitis was a NE, with RR 0.94 (95% CI 0.64–1.39).

#### SNRIs trials

Following headache and nausea, abdominal pain upper, dizziness, and somnolence were the most common AE presented by patients of non-active groups (see Table S2 in Supplementary Material). Abdominal pain was a NE with a RR 0.89 (95% CI 0.54–1.44).

#### TCAs trials

Following headache and nausea, dizziness, dry mouth, and respiratory disorder were the most common AE presented by patients of non-active groups (see Table S2 in Supplementary Material). Headache and respiratory disorder were NEs, with RR 0.96 (95% CI 0.66–1.39) and 0.58 (95% CI 0.23–1.45), respectively. Of note, dizziness and dry mouth were significantly more common in the active treatment groups.

#### Efficacy analysis

**Figure 4:**
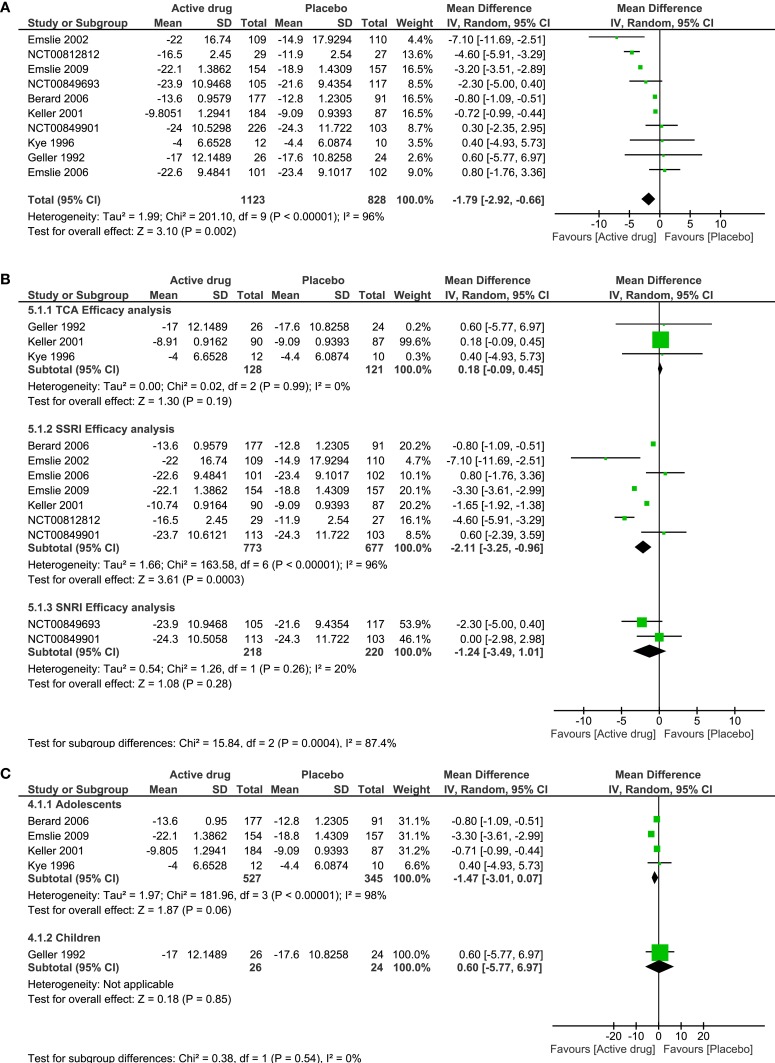
**Efficacy of antidepressant treatment**. **(A)** Forest plot of comparison: antidepressant versus placebo, outcome: efficacy. **(B)** Forest plot of subgroup analysis: efficacy by pharmacologic group of antidepressant versus placebo, and **(C)** Forest plot of subgroup analysis: efficacy by age group in antidepressant versus placebo. SD, standard difference; CI, confidence interval; TCA, tricyclic antidepressants; SSRI, selective serotonin reuptake inhibitors; SNRI, serotonin-norepinephrine reuptake inhibitors.

Ten out of 16 trials reported measures baseline and post-treatment for each intervention group. In all, 1123 patients in the active group and 828 in non-active group were included to calculate the pooled estimate of antidepressants efficacy. The heterogeneity for efficacy analysis was high (*I*^2^ = 96%), and there was a mild, but statistically significant reduction in the depression score scales in patients taking an antidepressant drug as compared to placebo, with the estimates in the same direction (SMD −1.79, 95% CI −2.92 to −0.66) (Figure 4A).

### Additional analysis

#### Subgroup analysis

In the analysis of pharmacologic drug group and risk of AE, there were no significant differences seen among a SSRI drug (RR 1.04, 95% CI 0.97–1.12) or a SNRI drug (RR 1.01, 95% CI 0.88–1.15) in comparison to non-active drug (Figure 3B). For patients taking a TCA and SNRI, there was no significant change in the depression severity scales compared to placebo (SMD TA 0.18, 95% CI −0.09 to −0.45; SMD SNRI −1.24, 95% CI −3.49 to −1.01). On the other hand, there was a significant reduction in the depression severity scales in patients taking a SSRI drug (SMD −2.11, 95% CI −3.25 to −0.96) (Figure 4B).

The subgroup analysis by age was only done for efficacy. We included four trials that were conducted solely in adolescents, and one trial that involved only children. There was no difference in the decrease of the depression scales in children or in adolescents who took an active drug in comparison to non-active, although adolescents showed a better improvement (SMD adolescents −1.47, 95% CI −3.01 to −0.07; SMD children 0.06, 95% CI −5.77 to −6.97) (Figure 4C).

#### Sensitivity analysis

For sensitivity analysis, we stepwise removed each trial in the AE analysis; neither of them exerted a significant change in the recalculated overall RR.

## Discussion

### Nocebo effect

Despite higher representation in placebo, both patients allocated to non-active and experimental groups had similar risk to develop AE. This has already been reported by other authors in pain-related trials (Amanzio et al., 2009; Hauser et al., 2012b; Mitsikostas et al., 2012) and clinical trials of depression in adults (Rief et al., 2009; Hegerl et al., 2010).

It should be noted that no RCTs included a no-treatment arm. As such, it is not possible to define whether adverse events are true NE, or manifestations of the disease itself. However, there are several indications that the reported adverse events are indeed of “nocebo” type. In previous systematic reviews on TCA trials, it has been shown that the most frequent nocebo symptoms were headache, nausea, dizziness, dry mouth palpitations, and tremor (Hazell and Mirzaie, 2013). In the current analysis of multiple types of antidepressants, we found the two most frequent instances of NE to be headache and nausea, where only nausea showed a significantly increased risk in placebo arms. The placebo group of TCAs trials was more likely to present dizziness and dry mouth, SNRIs upper abdominal pain and dizziness, and SSRIs nasopharyngitis and abdominal pain. The placebo groups in TCAs trials had the highest rate of NE, with nearly 33% of patients having stronger sedating and cholinergic profile, while placebo groups in the SSRI trials had the lowest rates. This is in accordance with the concept that the nocebo effect profile reflects those effects of active group (Gracely et al., 1985; Amanzio et al., 2009; Mitsikostas et al., 2012). Although our major findings about nocebo effect are similar than those of pain-related trials, it is imperative to highlight that a likely reason of these findings is insufficient descriptions, quality, and reliability AE assessment methods in the included randomized clinical trials.

Headache is commonly seen as a NE that affects compliance and adherence to treatments (Colloca and Miller, 2011; Mitsikostas et al., 2012). It is frequently presented in adolescents with depression (Nardi et al., 2013); however, reports show that healthy adolescents present headaches with prevalence of 11.9–29.1% (Ghandour et al., 2004; Bohman et al., 2012). Here, we found an 18% incidence of headaches among subjects assigned to placebo and 23% among patients assigned to an active drug, leading to reconsider its status as a real nocebo effect.

Many infection-related AE showed (non-significantly) higher frequency in the non-active group. This phenomenon might be explained by random chance or be related to the association between depression and altered immune function (Jones and Thomsen, 2013). Depression-induced immune system alterations, rather than nocebo, is a possible explanation.

Nocebo effect has been widely attributed to three important factors: expectations (Benedetti and Amanzio, 2013; Faasse and Petrie, 2013), suggestions (van Laarhoven et al., 2011), and conditioning (Hauser et al., 2012b; Data-Franco and Berk, 2013). It can be seen by the nocebo influence of clinicians, parents’ expectations, or patients sharing their experiences in the waiting room during visits. Moreover, anxiety-related symptoms of depression, somatization, or generalized psychological distress might influence the development of NE (Rogers, 2003). Bavbek et al. (2014) suggest that higher education and the patient history of hypersensitivity to medications increase the risk of nocebo responses. Less experienced physicians also appear to contribute to the nocebo response (Ashraf et al., 2014). Women may have a slightly larger nocebo response than men (Casper et al., 2001), as may homozygous carriers of the Val158 variant of the catechol-*O*-methyltransferase (COMT) gene (Val158met) (Wendt et al., 2014).

As suggested by Bingel (2014), some possible strategies to reduce NE include (a) optimizing treatment expectations and expectations of adverse effects, (b) a balanced presentations of risks and benefits, (c) teaching coping skills, (d) providing evidence-based information as opposed to alarmist web-forums, (e) better drug leaflet information, (f) hidden tapering in of medications, (g) pretreatment with drugs with low adverse effects, (h) video clips with patient examples coping well with adverse effects, (i) authentic and empathic patient–physician communication, (j) adequate information regarding disease, diagnoses, treatments, and adverse effects, (k) systematic feedback to patients, (l) pedagogical teaching (such as proactive check-back questions) to prevent negative biases and misunderstandings, and (m) adequately addressing patients’ anxieties, concerns, and expectations.

It is important to consider these factors in clinical and research practice in order to control this phenomenon. As the “therapeutic ritual” play an important role for the placebo and nocebo mechanism (Benedetti, 2012), physicians should cultivate positive reinforcement and continuous support to ensure patients’ adherence to the procedure and minimize the likelihood of nocebo (Benedetti and Amanzio, 2011).

Some authors have made some recommendations to reduce the nocebo effect in RCT’s. For instance, Amanzio (2011) have proposed the use of the natural history group as a “third arm” in pain trials, allowing the comparison of placebo groups with non-treated groups in order to seek for specific nocebo symptoms. Moreover, Cohen (2014) has proposed new approaches in the informed consent of trials that aim to avoid the nocebo influence due the advertisement of potential adverse events. Although these options could address the NE in clinical trials, this may be unfeasible due the critical ethical component of failing to inform a patient clearly about the procedures, known risks, and consequences, as well as the fact that untreated patients suffer from a serious condition.

### Efficacy of antidepressants

Currently, fluoxetine is the only antidepressant supported by the FDA for treatment of MDD in children aged 8 years and older. Also, our analysis indicates that fluoxetine has higher improvement on depression scales over placebo. However, the clinical status of fluoxetine use on pediatric patients remains in constant discussion due the incidence of suicidal ideation and suicidal attempts shown in previous clinical trials (Hetrick et al., 2012). In this order, the current recommendation is a constant monitoring of AE in patients taking SSRIs (Silva and Sampaio, 2011).

The efficacy analysis of antidepressants in depressive C&A when compared with placebo shows a mild statistically significant reduction in the depression scales used to determine the effect of treatment, which stood out in the Emslie et al. (2002) RCT (active drug was fluoxetine) due its marked efficacy over placebo (mean difference of −7.10 with 95% CI −11.69 to −2.51). Furthermore, the subgroup analysis of pharmacologic drug efficacy of antidepressants indicated that only SSRIs did significantly better than placebo, compared with SNRIs and TCAs. In particular, the current study findings indicate that fluoxetine has higher improvement on depression scales over placebo, consistent with the general use of fluoxetine as the primary treatment choice for C&A depression (Raz, 2006).

Regarding the treatment of the depressive population below 19 years old, there remains a discussion concerning the potential risk-benefit of SSRI treatment. Symptoms such as suicide ideation and suicide attempt may be considered as an inherent AE of these medications, a nocebo effect, or even due to the nature of the disease. Furthermore, Silva and Sampaio (2011) have proposed that these AE could be product of an underlying bipolar disorder in C&A undergoing treatment for MDD. In this order, the current recommendation is a constant monitoring of AE in patients taking these SSRIs.

An important concern that arises due to the lack of a well-known safe and effective treatment for C&A with MDD is the high rate of off-label prescription of antidepressants in this population. Compared with patients aged between 19 and 24 years, Czaja and Valuck (2012) found that patients under 18 had a higher number of prescriptions without strong support from the FDA. Antidepressants in C&A is less effective than the response obtained in adult patients, both in terms of treatment response and AE profile.

#### Assessing the heterogeneity of the clinical trials

The heterogeneity assessment showed homogeneous results for the pooled effect size of AE (*I*^2^ = 0, Figure 3). The explanation is that risks for AE were similar across the trials. In contrast, high heterogeneity was demonstrated in the efficacy analysis (*I*^2^ = 96%, Figure 4A). Heterogeneity for the efficacy analysis was related with the high variance of the pooled effect size in the improvement of depression scales: in TCA trials, placebo did better, while SSRIs did better than placebo.

We showed robustness of the results with the sensitivity analysis. After the exclusion of any trial from the analysis, the overall result did not modify significantly, neither of AE for efficacy analysis.

### Limitations

Our study has several limitations: first, attempts to obtain missing data were unsuccessful. Second, there was an inconsistency on adverse events’ assessment and report across the studies, with less than half of trials reporting the number of patients who presented AE, and without information about severity and about a potential relation between the symptom and the treatment, which constituted a constraint to perform the quantitative analysis for adverse events. Moreover, studies do not specify the type of AE assessment method used according to the nature of the symptom presented and studies used multiple methods for AE assessment, likely leading to different results. For instance, the structured approach was the most frequent assessment method for AE in RCTs, using scales, checklists, and questionnaires that led to only look for a specific set of AE. Others used a combination of methods, including clinician observation and patient spontaneous report, which might lead to a biased overestimation or underestimation of AE and decrease the quality of the adverse events reported. Third, the high proportion of unclear and high risk of bias within and across studies is concerning. Lack of information regarding the randomization, blinding process, and few details about the allocation concealment sequence might increase the probability of foreseeing the allocation group of participants and skew the primary outcome of the included trials (Schulz and Grimes, 2002). Finally, there were high rates of dropouts and withdrawals leading to a high risk of attrition bias, but none of the included studies described completely the frequency and reasons of dropouts in each intervention group, which did not allow to perform a deep analysis of dropouts in those patients allocated to active or control arms.

## Conclusion

The frequency of adverse effects did not differ between active treatment and placebo arms; moreover, the benefits of depressive treatment are mild and vary between studies. Our findings highlight the similar risk of presenting AE for patients receiving placebo compared to antidepressant treatment, indicative of (a) the relative safety of antidepressants and (b) possible NE.

### Implications for practice

Analysis of antidepressants’ efficacy for C&A showed an improvement of depression scales in patients taking SSRIs. Although SSRIs are the first line of pharmacological treatment for depressive C&A, there still remains a gap of knowledge regarding the safety profile due to suicidal-related attempts. For this reason, it is imperative to keep a constant monitoring of adverse events in patients undergoing SSRI treatment.

Our findings highlight the similar risk of presenting AE for patients receiving placebo compared to antidepressant treatment. It is of note that in clinical context, as well as in the research arena, there are some aspects to identify and address in depressive C&A in order to decrease the NE. The influence of parents’ expectations, anxiety-related symptoms of depression, and “therapeutic rituals” might influence the nocebo phenomenon.

### Implications for research

The influence of NE over the adverse events’ profile of antidepressants still remains doubtful, and it may be necessary to conduct further studies that allow understanding of the nature of nocebo phenomenon, perhaps also with no-treatment arms. For future studies, it is necessary to implement standardized methods to assess and report AE in RCTs of antidepressants in C&A.

It is imperative to clearly report methodological aspects of clinical trials in order to reduce the risk of bias related to randomization process, blinding of personnel and participants, allocation concealment method, and the report of dropouts and reasons for them. Strengthening these efforts should assure readers a complete understanding of the methodology and the quality of the results.

## Author Contributions

Dr. Rojas-Mirquez and Dr. Rodriguez-Zuñiga had full access to all of the data in the study and take responsibility for the integrity of the data and the accuracy of the data analysis, and also have contributed equally to the work. *Study concept and design*: Dr. Linnman, Dr. Borsook, Dr. Becerra, Dr. Rojas-Mirquez, Dr. Rodriguez, Dr. Bonilla-Escobar, and Dr. Garcia-Perdomo. *Acquisition of data*: Dr. Rojas and Dr. Rodriguez. *Analysis and interpretation of data*: Johanna Carolina Rojas-Mirquez, Research Fellow in Anesthesia at Harvard Medical School; Milton Rodriguez-Zuñiga, Research Fellow in Anesthesia at Harvard Medical School; Francisco Bonilla-Escobar, Research Associate at Instituto Cisalva, School of Public Health, Cochrane Center at Universidad del Valle, Cali, Colombia; Herney Garcia-Perdomo, Researcher and Professor, Cochrane Center at Universidad del Valle, Cali, Colombia. *Drafting of the manuscript*: All authors. *Critical revision of the manuscript for important intellectual content*: Dr. Bonilla-Escobar, Dr. Garcia-Perdomo, Dr. Borsook, Dr. Linnman, Dr. Rojas-Mirquez, Dr. Rodriguez-Zuñiga, and Mr. Mike Petkov. *Study supervision*: Dr. Bonilla-Escobar, Dr. Garcia-Perdomo, Dr. Borsook, and Dr. Linnman.

## Conflict of Interest Statement

The authors report no biomedical financial interests or potential conflicts of interest, neither have published nor submitted any related paper from this study.

## Supplementary Material

The Supplementary Material for this article can be found online at http://www.frontiersin.org/Journal/10.3389/fnbeh.2014.00375/abstract
