# Supplementary material for: Nocebo Effect in Randomized Clinical Trials of Antidepressants in Children and Adolescents: Systematic Review and Meta-Analysis
Source: Front Behav Neurosci. 2014 Nov 3;8:375. doi: 10.3389/fnbeh.2014.00375 (PMC4217505; doi:10.3389/fnbeh.2014.00375)
Supplement: Supplementary file 1 [file Data_Sheet1.ZIP › Supplementary Material.DOCX]

Supplemental Material, Online-only Material Document

**Nocebo effect in randomized clinical trials of antidepressants in children and adolescents: systematic review and meta-analysis.**

Content

1. e.Table 1. Search strategies for databases included in the review.
2. eFigure 1. Risk of bias summary for included studies.
3. eTable 2. Quality assessment on conduct and report of adverse events.
4. eFigure 2. Forest plot of comparison: adverse events by symptoms in antidepressants vs. placebo groups. A) Headache, B) nausea, C) Dizziness, D) Abdominal pain, E) Vomiting, F) Insomnia, G) Somnolence, H) Decrease appetite.
5. eTable 3. Adverse events in patients allocated in SSRI, SNRI and TCA groups. (SSRI, Selective serotonin reuptake inhibitors; SNRI, Serotonin–norepinephrine reuptake inhibitors; TCA, Tricyclic antidepressants).
6. **e.Table 1.** Search strategies for databases included in the review.

| **Database** | **Term used** | **Search Strategy** |
| --- | --- | --- |
| 1. PubMed | Medical Subject Headings Terms   - Condition: unipolar depression/ Depressive Disorder, Major/Major Depressive Disorder. - Intervention: antidepressant drug/antidepressant agents/antidepressants/ Antidepressive Agents, Second-Generation/ Antidepressants, Atypical/ Atypical Antidepressants/ Antidepressive Agents, Tricyclic/ Antidepressant Drugs, Tricyclic/ Antidepressants, Tricyclic. - Outcome: adverse effects/side effects/injurious effects/undesirable effects. | (unipolar depression OR Depressive Disorder, Major OR Major Depressive Disorder)) AND (antidepressant drug OR antidepressant agents OR antidepressants OR Antidepressive Agents, Second-Generation OR Antidepressants, Atypical OR Atypical Antidepressants OR Antidepressive Agents, Tricyclic OR Antidepressant Drugs, Tricyclic OR Antidepressants, Tricyclic)) AND (adverse effects OR side effects OR injurious effects OR undesirable effects) Filter: publication type: clinical trial + age: Child: birth-18 years From 1974/01/01 to 2013/12/31. |
| 2. CENTRAL | MESH Terms   - Condition: unipolar depression/ depressive disorder, major/ major depressive disorder. - Intervention: antidepressant drug/ antidepressant agents/ antidepressant/ Antidepressive Agents, Second-Generation/ Antidepressants, Atypical/ Atypical Antidepressants/ Antidepressive Agents, Tricyclic/ Antidepressant Drugs, Tricyclic/ Antidepressants, Tricyclic. - Outcome: adverse effects/ side effects/ injurious effects/ undesirable effects. - Population: child/ children/ adolescents. | #1: (unipolar depression OR Depressive Disorder, Major OR Major Depressive Disorder) AND (antidepressant drug OR antidepressant agents OR antidepressants OR Antidepressive Agents, Second-Generation OR Antidepressants, Atypical OR Atypical Antidepressants OR Antidepressive Agents, Tricyclic OR Antidepressant Drugs, Tricyclic OR Antidepressants, Tricyclic) AND (adverse effects OR side effects OR injurious effects OR undesirable effects)  #2: (child OR children OR adolescents)  #1 AND #2  Filter: TRIALS. |
| 3. EMBASE | Emtree Terms   - Condition: Major Depressive Disorder/Depressive disorder/ unipolar depression. - Intervention: antidepressants/ Antidepressive Agents/ antidepressant drug/Atypical Antidepressants/Second-Generation Antidepressive Agents/Tricyclic Antidepressive Agents. - Outcome: Side effect/ Adverse drug reaction/ Drug toxicity. | (Major AND depressive AND 'disorder'/syn AND 'antidepressant'/syn AND 'drug'/syn AND adverse AND effect) AND [randomized controlled trial]/lim AND [article]/lim AND ([child]/lim OR [adolescent]/lim) AND [humans]/lim AND [1974-2013]/py. |
| 4.BIREME | DeCS (in Spanish: “Descriptores en Ciencias de la Salud”) Terms-english   - Condition: Depressive Disorder/Major depressive disorder. - Intervention: Antidepressive Agents/Antidepressants/ Antidepressive Agents, Second-Generation/Antidepressants, Atypical/Atypical Antidepressants/ Second-Generation Antidepressive Agents/ Antidepressive Agents, Tricyclic/ Antidepressants, Tricyclic/ Tricyclic Antidepressive Agents. - Outcome: Drug Toxicity/Adverse Drug Reaction. | (depressive disorder OR depressive syndrome OR unipolar depression) AND (antidepressant drug OR antidepressant agents OR antidepressants) AND (Drug Toxicity OR Adverse Drug Reaction). |
|  | DeCS Terms-spanish   - Condition: Trastorno Depresivo Mayor/ depression/ trastorno depresivo. - Intervention: Antidepresivos/ Agentes Antidepresivos/ Antidepresivos de Segunda Generación/ Antidepresivos Atipicos/ Agentes Antidepresivos de Segunda Generación/ Antidepresivos Tricíclicos/ Agentes Antidepresivos Tricíclicos. - Outcome: Toxicidad de Medicamentos/ Reacción Adversa al Medicamento. | Trastorno Depresivo Mayor OR depression OR trastorno depresivo) AND (Antidepresivos OR Agentes Antidepresivos) AND (Toxicidad de Medicamentos OR Reacción Adversa al Medicamento). |
|  | DeCS Terms-portuguese   - Condition: Transtorno Depressivo Maior/Depressão Unipolar/Síndrome Depressiva. - Intervention: Antidepressivos/ Agentes Antidepressivos/ Antidepressivos de Segunda Geração/ Antidepressivos Atípicos/ Agentes Antidepressivos de Segunda Geração/ Antidepressivos Tricíclicos/ Agentes Antidepressivos Tricíclicos. - Outcome: Toxicidade de Drogas/Reação Adversa ao Medicamento. | (Transtorno Depressivo Maior OR Depressão Unipolar OR Síndrome Depressiva) AND (Antidepressivos OR Agentes Antidepressivos OR Timoanalepticos) AND (Toxicidade de Drogas OR Reação Adversa ao Medicamento) |

1. **eFigure 1.** Risk of bias summary for included studies.


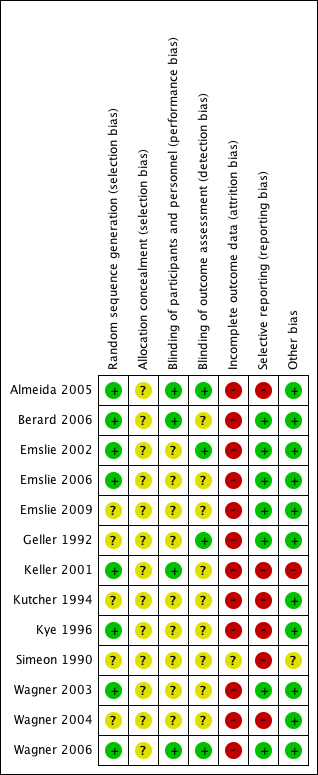


**Footnote:** + indicate low risk, - indicate high risk, ? indicate unclear risk**.**

1. **eTable 2.** Quality assessment on conduct and report of adverse events.

| **Article** | **Assessment on conduct** | | **Assessment on reporting** | | | | |
| --- | --- | --- | --- | --- | --- | --- | --- |
|  | Are definitions given of reported adverse effects? | How were adverse effects data collected: | Were any patients excluded from the adverse effects analysis? | Did the report give numerical data by intervention group? | Which categories of adverse effects do the investigators report? | Did the investigators report on all important or serious adverse effects, and how were these defined? | Were the methods used for monitoring adverse effects reported? |
| **Simeon 1990** | No. | No information. | Unclear. | No. | Most frequent symptoms. | No. | No. |
| **Geller 1992** | No. | Structured: weekly measure of AE with the Asberg Side Effects Scale. | Yes. | Yes. | By symptoms. | Yes, but they did not define them. | Yes. |
| **Kutcher 1994** | No. | Structured: weekly assess with a Side Effects Scales. | No. | No. | By scale scores, cardiovascular side effects and major adverse side effects. | No. | Yes. |
| **Kye 1996** | No. | Structured: through a Side Effects Scale. | Yes. | No. | Unclear. | No. | Yes. |
| **Keller 2001** | No. | Observations: adverse events were determined at each weekly visit. | No. | Yes. | By systems and symptoms. | Yes. | No. |
| **Emslie 2002** | Yes. | Combination: spontaneous report and Routine monitoring with a patient checklist. | No. | They report numerical data just for serious adverse events. | By symptoms: according to the treatment emergent (solicited and non -solicited) and severity of serious adverse events. | Yes. | Yes. |
| **Wagner 2003** | No. | Combination: spontaneous report and observation by investigators. | No. | Yes. | By symptoms and serious adverse events. | Yes. | Yes. |
| **Wagner 2004** | No. | Combination: Spontaneous report and observation by investigators. | No. | Yes. | By symptoms | Yes. | Yes. |
| **Almeida 2005** | No. | Structured: through the RECA scale. | No. | No. | Authors report the total score of RECA between groups. | No. | Yes. |
| **Wagner 2006** | No. | Combination: spontaneous report and observation by investigators. | No. | Yes. | By suicide related symptoms and serious. | Yes. | Yes. |
| **Berard 2006** | No. | Structured: at every visit by AE monitoring and vital sign determination. | No. | Yes. | By symptoms: according to the severity (mild, moderate and serious) | Yes. | Yes. |
| **Emslie 2006** | No. | Spontaneous report by patients. | Yes. | Yes. | By symptoms. | Yes, but they did not define them. | Yes. |
| **Emslie 2009** | No. | Combination: spontaneous report and observation by investigators. | No. | Yes. | By symptoms: serious and most frequent AEs. | Yes. | Yes. |

1. **eFigure 2.** Forest plot of comparison: adverse events by symptoms in antidepressants vs. placebo groups. A) Headache, B) nausea, C) Dizziness, D) Abdominal pain, E) Vomiting, F) Insomnia, G) Somnolence, H) Decrease appetite.


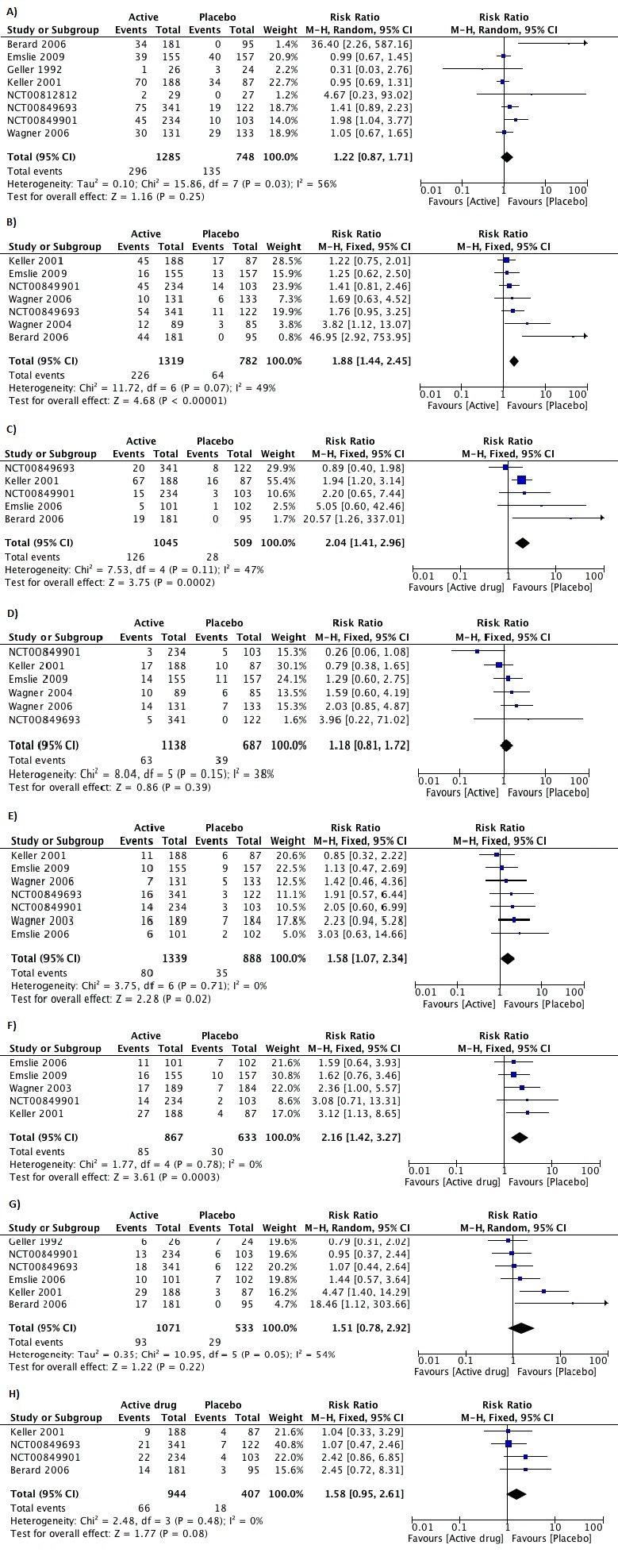


**Footnote:** (CI, Confidence interval).

1. **eTable 3.** Adverse events in patients allocated in SSRI, SNRI and TCA groups. (SSRI, Selective serotonin reuptake inhibitors; SNRI, Serotonin–norepinephrine reuptake inhibitors; TCA, Tricyclic antidepressants).

See the supplemental material file (Excel file).

**Footnote:** (* p-value less than 0.05; RR, Relative risk; CI, Confidence interval).
